# Supplementary material for: High prevalence of non-communicable diseases among key populations enrolled at a large HIV prevention & treatment program in Kenya
Source: PLoS One. 2020 Jul 2;15(7):e0235606. doi: 10.1371/journal.pone.0235606 (PMC7332043; doi:10.1371/journal.pone.0235606)
Supplement: Attachment — Submitted filename: Response to Reviewers_04_24_2020.docx [file pone.0235606.s001.docx]

**Response to Reviewers' Comments:**

**Reviewer #1:**

**This paper shows an interesting topic and information on NCD among key population living with HIV in Kenya. It shows a relatively high prevalence of NCDs in this population. The data is valuable but some aspects of the analysis and presentation of results are very unclear to me at present and require substantial improvement. I am also not convinced yet why the data cannot be share for publication with the reason explained.**

*Response: We wish to thank the reviewer for this comment. We wish to confirm to the reviewer that we have made every effort to make our analysis and presentation of the data as transparent as possible. Following PLOS’s data privacy policy and declaration of Helsinki on protecting vulnerable populations, we are under ethical obligation to safeguard the identity of our study participants. Further, in Kenya, key populations are in conflict with the law, and efforts to recognize them have been vehemently thwarted. Under protocol P258/09/2008, the Kenyatta National Hospital – University of Nairobi (KNH-UON) Ethics Research Committee has imposed restriction on the access of this data citing that sharing would be deemed to increase the risks or affect the safety or welfare of study participants.* However, w*e wish to confirm that data access can be granted upon request to the Secretary at KNH-UoN Ethics and Research Committee (*[*uonknh_erc@uonbi.ac.ke*](mailto:uonknh_erc@uonbi.ac.ke)*) for researchers who meet the criteria for access to confidential data.*

**Overall comments:**

**Reviewer Comment 1- You may read the submission guideline of Plos One and adjust your manuscript organization as suggested such as page and line number, abbreviation used in abstract, and then resubmit it**

*Response: We thank the reviewer for making this observation. In our resubmission, we have carefully followed PLOS One’s submission guidelines to reformat the manuscript, addressing its organization, page and line numbers. We have also spelled out abbreviation used in the abstract at the first instance that they are used.*

**Reviewer Comment 2- I feel hard to give the specific comment because the manuscript misses the line number in page 1 to page 13**

*Response: We have since reformatted the resubmitted manuscript and now have line numbers for pages 1 to 13.*

**Specific comments:**

**Reviewer Comment 1- I think the title of the paper is interesting. However, you may consider making it simpler and easier to catch up.**

*Response: We take note of the reviewer’s suggestion. Our updated title now reads “High prevalence of noncommunicable diseases among Key populations enrolled at a large HIV prevention and treatment program in Kenya”.*

**Reviewer Comment 2- In your abstract you may consider to used less abbreviation as suggested by the submission guideline of Plos One**

*Response: As per the reviewer’s comment and Plos One submission guideline, we have reduced the number of abbreviations in the abstract. Additionally, we have spelled out each abbreviation at the first instance it is used.*

**Reviewer Comment 3- You used the team "HIV-infected Key Populations" in some lines it make reader confused. You may use the other term such as key population living with HIV or HIV positive key populations.**

*Response: We apologize for any confusion that may have arisen as a result of using the term “HIV-infected Key populations”. We have since updated the manuscript using the term “key populations living with HIV”.*

**Reviewer #2:**

**Minor revisions to be made:**

**Reviewer Comment 1. The abstract could be divided in sections to make it easier to read**

*Response: We have since divided the abstract into sections improving its readability.*

**Reviewer Comment 2. Among the limitations in the discussion section, maybe you could add something about the fact that it was not possible to diagnose hypertension according to international recommendations (3 measurements at the same time, etc...).**

*Response: We thank the reviewer for this suggestion. We have included a sentence and a reference to reflect this limitation. The sentence (lines 358-359) reads “Similarly, our detection of hypertension based on updated guidelines that require a 24-hour mean blood pressure reading was limited owing to operational challenges at SWOP clinics .”*

**3. I would suggest to add some other interesting papers to the references list, as:**

**a. Ibrahim MM, Damasceno A. Hypertension in developing countries. The Lancet. 2012;380(9841):611-9.**

**b. Mbanya JC, Squire S, Cazap E, Puska P. Mobilising the world for chronic NCDs. The Lancet. 2011;377(9765):536-7.**

**c. Bloomfield GS, Hogan JW, Keter A, Sang E, Carter EJ, Velazquez EJ, et al. Hypertension and Obesity as Cardiovascular Risk Factors among HIV Seropositive Patients in Western Kenya. PLOS ONE. 2011;6(7):e22288.**

**d. Ciccacci F, Tolno VT, Doro Altan A, Liotta G, Orlando S, Mancinelli S, et al. Non communicable diseases burden and risk factors in a cohort of HIV+ elderly patients in Malawi. AIDS Res Hum Retroviruses. 2019.**

*Response: We thank the reviewer for the suggested references. We have since updated our references with three of the four journal articles.*

**Reviewer #3:**

**This is great piece of work given the attention that HIV/NCD comorbidity is receiving currently especially in countries with high HIV burden undergoing rapid epidemiological transition. The manuscript presents a clear and transparent research process with results emanating from appropriate analyses. However, the author is advised to consider making the following changes to improve manuscript readability and technical soundness.**

**Reviewer Comment 1: Table headings ought to be in uniform format. Table 1 and Table 2 headings appear to have inconsistent formats.**

*Response: We thank the reviewer for their comments. We have formatted the table headings and now have a consistent format from Table 1 to 4.*

**Reviewer comment 2: Table 2 column N should be described either by a footnote or by column heading to avoid confusing the reader with another N=271. Table 3 footnotes should be numbered consecutively:1,2,3 and not 2,2,3.**

*Response: We have updated the column labelling to consistently reflect a common “N” that is unambiguous. We have also updated the consecutive numbering of footnotes on Table 3.*

**Reviewer Comment 3: Could you please make the last columns for Table 2 wider in order to cover confidence intervals in one line other than two lines as is the case now? This would help your Table 2 look tidier.**

*Response: We oblige and have reformatted Table 2 to cover confidence intervals in one line.*

**Reviewer Comment 4: The sentence in line 53 could read better if you removed "albeit" and replaced "comparable" with "comparably".**

*Response: As per the reviewer’s suggestion, we have removed the word “albeit” on line 53 and replaced “comparable” with “comparably” to improve its readability.’*

**Reviewer Comment 5: Please interpret statistically significant odds ratios in univariate analyses. Also in line number 35,"unadjusted model" should be changed to unadjusted analyses or univariate analyses since this is not one model perse, all variables assessed in univariate analyses represent individual univariate logistic models.**

*Response:* Thank you for pointing this out. We have provided an interpretation of the significant odds ratios from univariate analysis and now refer to univariate analyses in line 256 - 257 as opposed to “unadjusted model”.

**Reviewer Comment 6: A sentence in line 37 reads "When the model was adjusted, all prior significant associations between NCD diagnosis and increased age, unemployment status, BMI and CD4 ceased" Consider changing this sentence to "even though increased age, unemployment status, BMI and CD4 were associated with NCD diagnosis in unadjusted analyses, they were not significantly associated with NCD diagnosis in the multivariate, adjusted analyses".**

*Response: As per the reviewer’s suggestion, we have since updated the sentence in line 258 to read as follows "* *Even though increased age, BMI and CD4 were associated with NCD diagnosis in unadjusted analyses, significant association with NCD diagnosis in the adjusted analyses remained only for categories of BMI 30 kg/m2 and above, and ages 45 years and above (borderline statistically significant)".*

**Reviewer Comment 6 part B: The authors should also explain why variables with P> 0.2 such as sex, alcohol use and smoking were included in the multivariate model as this is not consistent with their analysis plan in which an automated stepwise backward logistic regression approach was selected to build a multivariate model to determine predictors for NCD prevalence from independent predictors of NCDs with a p-value of 0.2 or less in univariate analyses.**

*Response: Apologies for the deficiency in description of our methods**. Age, sex, alcohol use and smoking were considered apriori as potential confounders of the association of studied risk factors with NCD and included in the final multivariate logistic regression model. We have updated the statistical analysis section accordingly, line 118-119.*

**Reviewer Comment 7: The author should consider adding references to his claim in line number 89 "Studies have [90] documented harmful consumption of alcohol, smoking tobacco, illicit drug use, and risky sexual behaviors [91] among KPs as factors that increase their risk for NCDs."**

*Response: As per the reviewer’s comment, we have included three references that support the claim on documented harmful consumption of alcohol, smoking tobacco, illicit drug use, and risky sexual behaviors [91] among KPs as factors that increase their risk for NCDs.*

**Reviewer Comment 8: The author tries to contradict earlier research findings that they did not find an association between ART use and NCD prevalence in line number [66]. This should be avoided as the study was not designed to show association between ART use and NCD prevalence since the study enrolled no participants without exposure to ART. On the same note, the author also reports lack of association between NCD and detected viral load. In this study, at no point were viral load measurements reported. Therefore, such claims are not supported and should be removed from the manuscript.**

*Response: We thank the reviewer for this suggestion and have expunged the line 66 at the end of paragraph two in the discussion section. Indeed, our study did not enroll participants with no ART exposure therefore unable to make the claim of an association between ART use and NCD prevalence. We have also removed any mention of an association with viral load measurements in our study since these were not presented in this manuscript.*

**Reviewer Comment 9: The author should remove any references in the conclusion section of the manuscript.**

*Response: We have removed all references in the conclusion section.*

**Reviewer #4:**

**The empirical analysis is competent, and the authors reference much of the relevant literature. A review on NCDs among key populations (KPs) is generally valuable, in light of concerns on the burden of NCDs among PLWH in general and specifically of the role and specific needs of key populations.**

**My reservations on publication of the paper in its present form primarily regard two aspects:**

**First, does the paper provide an analysis on NCDs among KPS? Not really. 94 percent of the sample are FSWs, and only 6 percent (n=86) MSMs. 86 percent of the cases of NCDs (total NCDs: n=271) are instances of high blood pressure (n=233), and the number instances of chronic respiratory disease (n=16) or cancers (n=10) do not allow a substantial empirical analysis. Against these numbers, is puzzling why much of the paper is cast in terms of "NCDs" among KPs – it really is about high blood pressure among FSWs, and – because implications and determinants of NCDs arguably differ – focusing the empirical analysis on instances of any NCD blurs the lessons which could be learned.**

*Response:* Thank you for the thoughtful comment. We utilized data from a routine HIV prevention and treatment program serving the two key populations. As such, the distribution of attended population and outcomes of interest was analyzed as-is. The reviewers rightly points out this and as part of the paper we now acknowledge this limitation in the discussion and have reframed our interpretation in this light. Although the paper highlights the prevalence of key NCDs in group of female and male sex workers enrolled in a funded HIV prevention and treatment program in Nairobi Kenya, majority of those with conditions of interests were women. Sex work and same sex relationships are still illegal and stigmatized in Kenya. However, females who sell sex seem to be more tolerated than MSM engaged in the trade. The latter suffer double stigma and their position is even worsened by being HIV infected. Therefore, females were over represented in the study sample as fewer men out of the many closeted MSM have taken that leap of faith to enroll in the ongoing HIV prevention and treatment programs. We acknowledge that the over representation of women in the sample limits the generalizability of results to key populations experiences in Kenya or the region. However, the data provides some insights into NCD burden in this marginalized population that has not been reported elsewhere.

**Second, are there any useful findings? Note sure – the results broadly mirror the empirical evidence on risk factors for hypertension – prevalence is increasing with age and with BMI. Other factors appear irrelevant (this could be tested more explicitly – do factors pervasive with respect to key populations play any role?). However, we would also want to understand whether prevalence of NCDs differs from prevalence in the general population. Doing such a comparison explicitly is beyond the scope of this paper (sample on KPs only), but the authors do not exhaust possibilities on comparing their findings with data on the general population (e.g., from DHS and related data). Relatedly, what is the relevance of the findings with regard to the management of HIV or NCDs among key populations?**

*Response:* Again, we greatly appreciate this insightful comment. While we do acknowledge the limitations of our study in this regard, we note that our recommendation to funders and policy makers to promote integration of NCD- HIV programming in the future is evidence based too. We have previously published NCD prevalence’s from the general population using similar routine HIV prevention and treatment data (ref Achwoka D, Waruru A, Chen T-H, Masamaro K, Ngugi E, Kimani M, et al. Noncommunicable disease burden among HIV patients in care: a national retrospective longitudinal analysis of HIV-treatment outcomes in Kenya, 2003-2013. 2019;19(1):372. doi: 10.1186/s12889-019-6716-2.). PLHIV in Kenya were found to have a high prevalence of NCD diagnoses with proportion of any documented NCD among PLHIV being 11.5% (95% confidence interval [CI] 9.3, 14.1).

**Minor points:**

**It is not clear on what basis variables have been excluded in the multivariate analysis (Table 4). Excluding ART regimen and prior TB history appears sensible (p-value>0.9 in univariate regression), but there are numerous other variables with p-values in the vicinity or 0.7 or 0.8 in the multivariate analysis which are included in the regression.**

*Response: Apologies for the deficiency in description of our methods. Age, sex, alcohol use and smoking were considered apriori as potential confounders of the association of studied risk factors with NCD and included in the final multivariate logistic regression model. We have updated the statistical analysis section accordingly.*

**Table 4: Review p-value of 1.11, BMI 30+ adjusted odds ratio.**

*Response:* This was an error. The correct p-value is 0.029.

**In a couple of places, I felt that the paper would benefit from a round of copy-editing to improve precision.**

*Response: We have revised the whole manuscript following PLOS One guideline and making the presentation as succinct as possible. The revised manuscript has a couple edits to that effect.*
