# Supplementary material for: High prevalence of non-communicable diseases among key populations enrolled at a large HIV prevention & treatment program in Kenya
Source: PLoS One. 2020 Jul 2;15(7):e0235606. doi: 10.1371/journal.pone.0235606 (PMC7332043; doi:10.1371/journal.pone.0235606)
Supplement: Attachment — Submitted filename: Response to Reviewers.docx [file pone.0235606.s002.docx]

**Response to Reviewers' Comments:**

**Reviewer #1:**

**Many thanks for your revision. I found a great improvement with the revised version. To move on further process, I am suggesting for a few more minor revisions as described below;**

**1- I think you should remove capital letter of “Key populations” in the title and a few other places in the manuscript**

*Response: We thank the reviewer for this suggestion. We have since removed the capital letter ‘K’ in key populations both in the title and in the manuscript text.*

**2- I think you should add in text citation and reference for first sentence in the second paragraph (line 51-53).**

*Response: As suggested by the reviewer, we have included an in-text citation and reference for the first sentence in the second paragraph.*

**3- I think you should use less human possessive term in the manuscript. You should make the language use to more academic. You should consider removing those words such as in line 72 “we only…”, line 107 “Our analyses”, line 136 “We analyzed”, line 216 “Our proxy”, line 270 “our study”, line 271 “In our study”, line 274 “ours among”, line 286, line 289, “in our study”, line 295 “we…” … “our”… please check for the rest and you may use term “this study” to replace “our study”…**

*Response: We continue to thank the reviewer for this important comment. The entire manuscript has been updated accordingly to rid it of human possessive terms and is now entirely in academic language.*

**4- Do you have any rational why age, gender, alcohol use and smoking were considered as priori as potential confounder? Any learning from other literature?**

*Response: We thank the reviewer for this query. The aforementioned factors met the criteria for confounding (being associated with both the risk factor of interest and the outcome, unequal distribution among comparison groups, and not being an intermediary step in causal pathway). We found these factors in our literature review and point the reviewer to the Kenya STEPwise survey for non-communicable risk factors 2015 Report.*

**5- In line 274, you mention “studies similar to ours among…” it seems does not fully completed yet.**

*Response: We realize that line 274 was unclear and have revised line 273-275 to improve clarity. It now reads “ Despite a heightened impetus to refocus on populations at increased risk for both NCDs and HIV infection , studies among key populations living with HIV remain rare [22]. That notwithstanding, systematic reviews outside SSA suggest that sexual minorities exhibit higher rates of NCDs [11]”.*

**6- At the end of the data collection or the starting of data analysis, you may add another sentence indicating that data is cleaned and imported into Stata for data analysis.**

*Response: We have incorporated this important suggestion in the manuscript under the sub-title ‘Study procedures and data collection’. It now reads* “*Data were cleaned and subsequently imported to STATA 15 (STATA Corporation, Texas USA) for data analysis”.*

**7- In the data analysis, you may need to also describe how will you report the result of of the analysis such mean, median, SD, 95% CI… and abbreviate any possible term here than you don’t have to write full word in the result (eg. in line 160, 95% confident interval (CI)).**

*Response: We thank the reviewer for this comment. We have now included a sentence within the statistical analyses to describe reporting of analyses such as mean, SD and 95% confidence intervals.*

**8- In line 136-138, I think this seem reported the data collection and analysis section. You may start directly reporting the result of analysis to be concise.**

*Response: We agree with the reviewer’s suggestion. We have since revised the lines 136-138 to avoid repetition with the data collection and analysis section. It is now concise.*

**9- In line 139, I think you can use abbreviation of FSWs and MSM as they been abbreviated already in the introduction. You may also need to check other line to make it consistent.**

*Response: We have updated the entire manuscript and checked for consistency as per the reviewer’s comment. Now the abbreviations of FSWs and MSM appear consistently.*

**10- Could you please add concrete list of inclusion / exclusion criteria for administer abstracted data? I found pieces of information from line 85 to 90 but not so convinced yet.**

*Response: We have bolstered this section as suggested by the reviewer and included both inclusion and exclusion criteria for the data abstraction. As part of inclusion we considered: a) age 15 and above; b) Enrollment into the SWOP clinics between periods October 2012 and September 2015; c) HIV positive at enrollment or seroconverted during the period of study and d) Identified as MSM or FSW as typology. For exclusion we considered: a) HIV negative key population; b) other key population typology – including PWIDs and transgender; c) key population enrolled outside the study period and d) key population under the age of 15 or missing information on age.*

**11****- I still would like to suggest, the author consider to discuss their finding with the other studies such as “High prevalence of non-communicable diseases and associated risk factors amongst adults living with HIV in Cambodia” and/or “Non-communicable diseases and related risk behaviors among men and women living with HIV in Cambodia: Findings from a cross-sectional study” because I found this study is quite similar in some setting this study participant are people living with HIV even it focus to key population.**

*Response: We thank the reviewer for this insightful suggestion. We note that we have taken due diligence and studied the two papers. We are happy to report that the findings from the two papers from Cambodia add value to our manuscript and have been included as part of our discussion. Specifically, they are referenced in the second paragraph in the discussion.*

**Reviewer #4:**

**Minor revisions to be made:**

**Reviewer Comment 1. The authors provide competent and diligent responses to reviewers' comments, specifically those by myself but also (according to a cursory overview) those from other reviewers.**

**One - I believe - important shortcoming remains. The authors do not provide a substantial discussion comparing NCDs among key populations (KPs) with the prevalence of NCDs in the general population or among PLWH overall. Understanding these differences, though, would be important for interpreting the findings and drawing policy-relevant conclusions. Are there factors specific to KPs driving high prevalence of NCDs? The findings suggest that this may not be the case, as pointers for risk behaviour come out largely insignificant (the statistically significant variables are age and BMI 30+). A pointer to the findings of the paper referred to in the response to comments (Noncommunicable disease burden among HIV patients in care: a national retrospective longitudinal analysis...), with some author overlap with the present paper, would also contribute to placing the findings of the present paper in context, and I find it puzzling to see that the authors do not make such connections.**

**This shortcoming may not preclude publication, as the analysis per se is competent, but the value of the paper is clearly diminished by the authors' reluctance to place their findings in this wider population or PLWH context.**

*Response: We are thankful to the reviewer for raising this important concern. We acknowledge the gravity of the reviewer’s sentiments and have bolstered our discussion substantially to include comparisons of NCDs between key populations and general population PLHIV. We have included a new paragraph in the discussion section (now appears as the second paragraph) and added three references. These references include studies from concentrated HIV epidemics in Cambodia, recent modeling of NCD burden among PLHIV and general population as well as our very own study among general population PLHIV. We believe this will offer justice and improve the relevance of our study for policy conclusions.*

**Reviewer Comment 2. On the editorial side, I sense that while the language is clear throughout, the paper would benefit from one round of professional copy-editing.**

*Response: We thank the reviewer for this suggestion. We have conducted additional copy editing and would like to assure both the reviewer and editor that we now have a high-quality product commensurate to PLOS one’s standards.*
